# Editorial: Ozone in medicine: Biochemical background, physiological modulation and clinical applications

**DOI:** 10.3389/fphys.2023.1112860

**Published:** 2023-02-06

**Authors:** Bernardino Clavo, Emma Borrelli

**Affiliations:** ^1^ Research Unit, Chronic Pain Unit, and Radiation Oncology Department, Hospital Universitario de Gran Canaria Dr. Negrín, Las Palmas, Spain; ^2^ Fundación Canaria Instituto de Investigación Sanitaria de Canarias (FIISC), Las Palmas, Spain; ^3^ Instituto Universitario de Enfermedades Tropicales y Salud Pública de Canarias de la Universidad de La Laguna, Tenerife, Spain; ^4^ Department of Medical Biotechnologies, University of Siena, Siena, Italy; ^5^ Postgraduate Course on Oxygen Ozone Therapy, University of Siena, Siena, Italy

**Keywords:** ozone therapy, oxidative stress, Nrf2, pain, chemotherapy-induced peripheral neuropathy (CIPN), rheumathoid arthritis, bronchial asthma

The triatomic allotrope of oxygen was described as “ozone” for the first time by Christian F. Schonbein in 1839, and it is the third most powerful oxidant agent after fluorine and persulfate (Bocci et al., 2009). On the basis of its strong oxidant capacity, the first use of ozone in medicine at the end of the XIX century was as large spectrum germicide, for example in the potabilization of drinking water (Tanner et al., 2004), and several potential clinical uses as widely described in the authoritative book by Bocci, (2011).

The goal of this Research Topic *“Ozone in Medicine: Biochemical Background, Physiological Modulation, and Clinical Applications”* is to offer a comprehensive picture of the new research evidence in ozone therapy from bench to bedside. In fact, the mini-review by Re, (2022), about the main action mechanisms of ozone, links the old knowledge about the role of ozone in modulation of oxidative stress with the recent findings about its modulation of the nuclear factor erythroid 2-related factor 2 (Nrf2). The review on pain management by Hidalgo-Tallón et al., based on the past, describes the clinical evidence about pain, one of the most evaluated symptoms treated with ozone. B Clavo et al. published a preliminary report where ozone is added in the management of painful chemotherapy-induced peripheral neuropathy (CIPN). This study confirms the efficacy of ozone therapy on pain relief and suggests a potential new indication for ozone application for a syndrome lacking successful therapies, thus opening a field for future research. The fourth article, by OS León Fernández et al., was focused on two different diseases in elderly patients mediated by oxidative stress, rheumatoid arthritis, and bronchial asthma.

Most of the potential clinical uses of ozone treatment are not based on the direct effect of the topical treatments. In fact, the majority of clinical studies with ozone treatment are carried out using systemic ozone treatment, principally by indirect venous administration or by rectal insufflations. Using these approaches, the effects of ozone are obtained in an “indirect way”, which resembles a concept described 2,000 years ago by Hippocrates, *vis medicatrix naturae*, which makes reference to the body’s own mechanisms that are within us, which try to restore our health and cure our diseases (Chu et al., 2022). By systemic treatment, ozone interacts with polyunsaturated fatty acids (PUFA), antioxidants, and other components of plasma (indirect venous administration) or glycocalyx and mucoproteins (rectal insufflation) to generate reactive species of oxygen (ROS) and lipid oxidation products (LPOs), which on turn interact with cell membranes (in blood cells or cells of the intestinal mucosa) and *via* the blood stream LOPs can reach distant tissues. These actions produce a controlled and transient oxidative stress that will induce an adaptive body response leading to an overall enhancement of antioxidant systems. The key role of this action of ozone depends on the regulation of Nrf2, a relationship described for the first time in the XXI century. Further details about the relationship of ozone with oxidative stress and its regulation of antioxidant mechanisms by the Nrf2 pathway were described in the review of Re, (2022). This way, the potential beneficial and deleterious effects of ozone should be similar to those described for Nrf2 activation (Milkovic et al., 2017; Rojo de la Vega et al., 2018; Borrelli, 2021).

**FIGURE 1:**
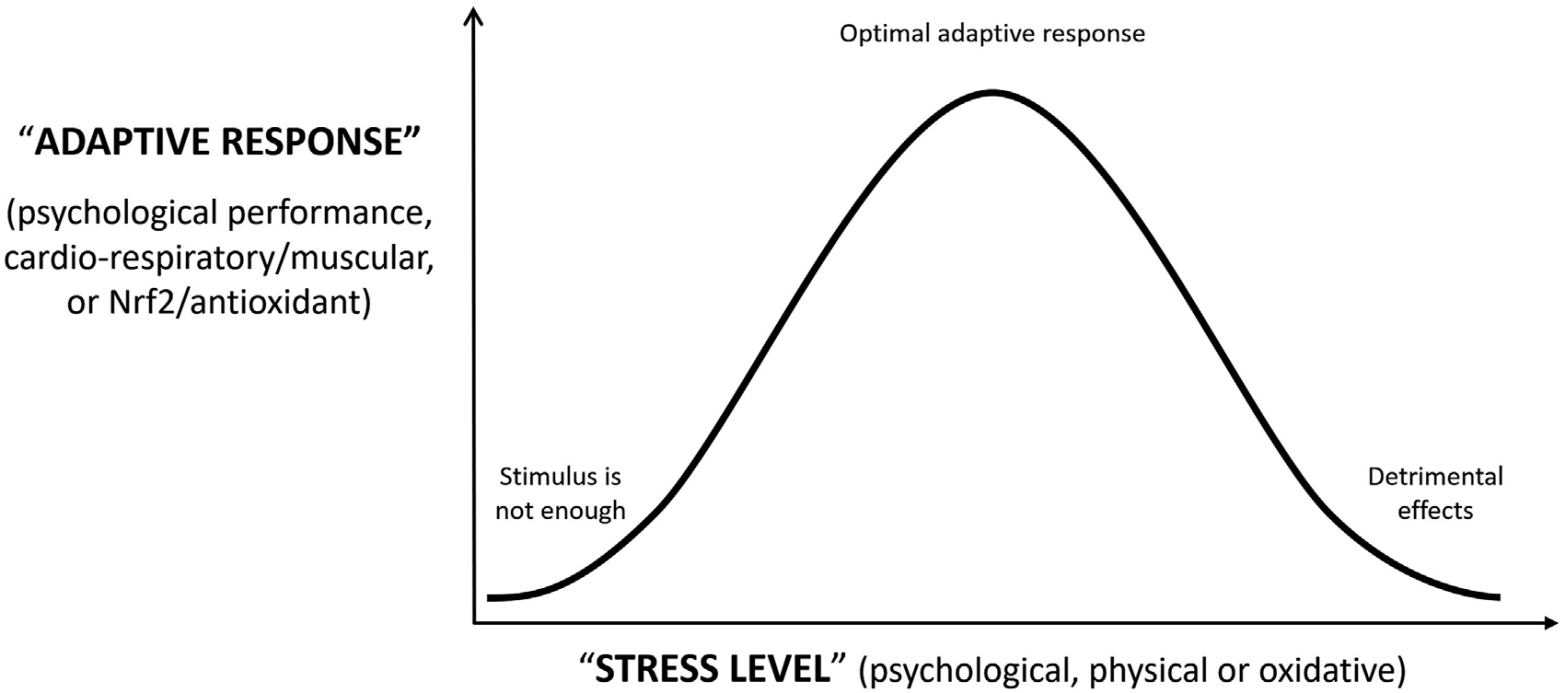
Adaptive response of the organism to psychological, physical or oxidative “stress level”.

It is interesting to note that the potential beneficial effect at low-dose and detrimental effect at high-dose (hormetic effect mentioned by L Re, (2022) has been described for ozone (Bocci et al., 2011) and Nrf2 (Calabrese and Kozumbo, 2021), and they remind us the inverted U-shape response hypothesis described by Yerkes-Dodson at the beginning of the XX century for the relationship between psychological stress and productivity (Yerkes and Dodson, 1908). This would be similar to the relationship between physical exercise and cardio-respiratory or muscular adaptive response, or the relationship between oxidative stress induced by ozone and Nrf2/antioxidant adaptive response (Borrelli, 2021). In all these situations, it exists an appropriate range of stimulation/stress (psychological, physical, or oxidant) which is able to induce a beneficial adaptive response but only up to a threshold level. Above that level, the stimulus/stress overwhelms the adaptive capacity leading to detrimental effects. On the other hand, very small stimulus/stress will be managed by the basal capacity without any adaptive response. (Figure 1).

Despite the well documented action mechanisms of ozone, well designed randomized controlled trials (RCT) are required. Although there are a limited number of RCT, fortunately, the number and clinical conditions evaluated are continuously growing.

Probably, the ozone-research field with wider and deeper clinical evidence is pain management. The article by Hidalgo-Tallón et al. describes RCTs, systematic reviews, and meta-analyses published about different pain syndromes treated by ozone as knee pathology (osteoarthritis, knee tendinopathies, patellofemoral chondromalacia), shoulder pathology (subacromial tendinopathy, calcifying tendinitis of the shoulder, and non-surgical lesions of the rotator cuff), disc herniation (and cervical and low back pain), rheumatoid arthritis among others. Overall, most studies described significant clinical improvement in treatment groups including ozone, alone or combined with conventional therapy. The most relevant clinical evidence is described for the treatment of knee osteoarthritis and lumbar disc herniation, although it is not accepted by many clinicians Hidalgo-Tallón et al. We hope that this will change soon in the interest of our patients.

A brief research report by B Clavo et al. extends the previous research field of ozone to a new painful clinical condition, the CIPN, which has been established as an urgent area of research by the American Society of Clinical Oncology (ASCO) (Markham et al., 2020). This is a very preliminary no-RCT study, with a very small sample size, and the encouraging results should be viewed with caution until larger RCTs have been conducted. However, the magnitude and length of the observed effects and the limited therapeutical approaches for CIPN (Zhang, 2021) support further research. Indeed, the authors have an ongoing RCT (NCT04299893).

The last research, by OS León Fernández et al., is a pilot study that integrates some characteristics of the three previous articles. On the one hand, this work is focused on the action mechanisms of ozone, with i) an assessment of nitric oxide (NO), prostacyclin, and thromboxane in elderly patients with bronchial asthma, ii) and an assessment of NO and several oxidative stress parameters in elderly patients with rheumatoid arthritis. On the other hand, patients with rheumatoid arthritis were studied inside a RCT, and it was also described the beneficial clinical effect of ozone in pain and in some scales of quality of life.

The use of ozone in medicine is based on many years of old experiences and clinical reports and new scientific principles. However, only rigorous continuous evidence-based work will dispel misconceptions and skepticism about this therapy.
